# The relation between socioeconomic and demographic factors and tumour stage in women diagnosed with breast cancer in Denmark, 1983–1999

**DOI:** 10.1038/sj.bjc.6603294

**Published:** 2006-08-08

**Authors:** S O Dalton, M Düring, L Ross, K Carlsen, P B Mortensen, J Lynch, C Johansen

**Affiliations:** 1 Department for Psychosocial Cancer Research, Institute of Cancer Epidemiology, Danish Cancer Society, Strandboulevarden 49, Copenhagen DK-2100, Denmark; 2 DBCG's Secretariat, Rigshospitalet 7003, 9 Blegdamsvej, Copenhagen DK-2200, Denmark; 3 National Centre for Register-based Research, Tåsingegade 1, Aarhus C DK-8000, Denmark; 4 Department of Epidemiology, Biostatistics and Occupational Health, McGill University, Montreal, Canada

**Keywords:** socioeconomic position, breast cancer, stage, population-based, registries

## Abstract

The authors investigated the association between socioeconomic position and stage of breast cancer at the time of diagnosis in a nationwide Danish study. All 28 765 women with a primary invasive breast cancer diagnosed between 1983 and 1999 were identified in a nationwide clinical database and information on socioeconomic variables was obtained from Statistics Denmark. The risk of being diagnosed with a high-risk breast cancer, that is size >20 mm, lymph-node positive, ductal histology/high histologic grade and hormone receptor negative, was analysed by multivariate logistic regression. The adjusted odds ratio (OR) for high-risk breast cancer was reduced with longer education with a 12% reduced risk (95% confidence interval (CI), 0.80,0.96) in women with higher education and increased with reduced disposable income (low income group: OR, 1.22; 95% CI, 1.10,1.34). There was an urban–rural gradient, with higher risk among rural women (OR 1.10; 95 % CI, 1.02, 1.18) and lower risk among women in the capital suburbs (OR, 0.85; 95% CI, 0.78, 0.93) and capital area (OR, 0.93; 95% CI, 0.84–1.02). These factors were significant only for postmenopausal women, although similar patterns were observed among the premenopausal women, suggesting a subgroup of aggressive premenopausal breast cancers less influenced by socioeconomic factors.

Although affluent women have a higher incidence of breast cancer than socially deprived women, several studies using individual or area-based socioeconomic measures have shown that deprived women with breast cancer have poorer survival from disease (Carnon *et al*, 1994; Stavraky *et al*, 1996; Kravdal, 2000; Bradley *et al*, 2001; Thomson *et al*, 2001; Menvielle *et al*, 2005; Woods *et al*, 2005).

Long-term prognosis of breast cancer patients strongly depends on stage of disease at the time of diagnosis and thus, social inequalities in tumour progression at the time of diagnosis which has been reported in several (Schrijvers *et al*, 1995; Catalano and Satariano, 1998; Lannin *et al*, 1998; Macleod *et al*, 2000; Bradley *et al*, 2001; Kaffashian *et al*, 2003; Schwartz *et al*, 2003; Adams *et al*, 2004; Davidson *et al*, 2005) but not all studies (Carnon *et al*, 1994; Arndt *et al*, 2001; Brewster *et al*, 2001; Liu *et al*, 2005; Robsahm and Tretli, 2005) could contribute to social inequality in survival. Further, a higher proportion of oestrogen receptor positive tumours among women with a higher socioeconomic position has been reported (Gordon, 1995; Twelves *et al*, 1998; Thomson *et al*, 2001); this could either be interpreted as a difference in time of diagnosis (Hellman, 1994; Zhu *et al*, 1997) or as a different distribution of high-risk and low-risk breast cancer types (Anderson *et al*, 2005) across socioeconomic groups.

We investigated the relation between socioeconomic position and tumour progression as measured by high-risk *vs* low-risk breast cancers at the time of diagnosis, stratified by menopausal status in a large nation-wide population based cohort of 28 765 women diagnosed with breast cancer in Denmark between 1983 and 1999.

## MATERIALS AND METHODS

### Case ascertainment

The study population consisted of all 31 770 women identified in the files of the Danish Breast Cancer Cooperative Group (DBCG) with a primary invasive breast cancer diagnosed between 1 January 1983 and 31 December 1999 and who were less than 70 years of age at the time of diagnosis. The DBCG has since 1977 registered breast cancer patients in Denmark and conducted protocol-based randomised trials of surgery, radiation, chemotherapy or endocrine therapy in patients with primary invasive breast cancer (Andersen and Mouridsen, 1988). The registry contains information on 95% of all Danish women below 75 years of age diagnosed with breast cancer over the period and each record contains information about prognostic factors: tumour size, histopathological grade, number of axillary lymph nodes removed, number of tumour-positive axillary lymph nodes, hormone receptor status, treatment modalities, adjuvant treatment and age at breast cancer diagnosis. The database holds continuously updated information on relapse-free interval and localisation of first recurrence.

All identified women with breast cancer were classified into a low-risk or high-risk group. The criteria for being a low-risk breast cancer patient has changed over the period and so we redefined a set of criteria consistent with the latest risk protocol to classify all women regardless of the protocol under which they were originally diagnosed and treated. Low-risk breast cancers were defined by tumour ⩽20 mm, no tumour positive axillary lymph nodes, grade of malignancy I or unknown or nonductal tumour, and receptor positive or unknown. Tumour size only reported as <50 mm (*N*=460) was considered as unknown in this study.

### Socioeconomic factors

Information on socioeconomic characteristics of women with breast cancer was obtained by data linkage to the population based Integrated Database for Labor Market Research (IDA) administered by Statistics Denmark since 1980 (Statistics Denmark, 1991). The core variables in the database are based on a linkage between all people in Denmark (5.4 million per January 2004), all companies with more than one employee (around 230 000), the taxation authorities and the Registry Relating to Unemployment and the Central Population Registry (Statistics Denmark, 1991). From IDA, we obtained information on the individual level about a number of demographic and socioeconomic variables for the end of the year of breast cancer diagnosis. Further, we used IDA to identify spouses, cohabiters and children aged 0–17 years of all women with breast cancer at the time of breast cancer diagnosis. A partner was defined as an unrelated person of the opposite gender, over the age of 16 years, with maximum 15 years of age difference living at the same address and with no other adult living there. Thus, in this study partner includes both those married and those cohabiting with the index-persons, whereas non-registered homosexual partners and partners with more than 15 years age difference were excluded. In total, we identified 22 313 partners and for each of these, we obtained the same socioeconomic and demographic information. From the Building and Dwelling Register which contains information on exact address codes on all Danish people, we obtained information on size, type and tenure of dwelling (Thygesen, 1995).

Highest attained education was categorized as basic school/high school, vocational training, higher education and unknown; job position as higher functionaries/self-employed, lower functionaries, skilled workers, unskilled workers, not in the work force (unemployed and other economically inactive – predominantly housewives) and pensioners (retirement and disability); disposable income adjusted for number of people in household ((household disposable income/number of people in household)^0.6^) and deflated according to the 2000 value of the Danish crown (DKK) was categorized as <100 000 DKK/year, 100 000–129 999 DKK/year, 130 000–165 000 DKK/year, >165 000 DKK/year; housing tenure as owner-occupied or rental; and size of dwelling as 0–99 m^2^, 100–124 m^2^, 125–149 m^2^, ⩾150 m^2^. Demographic variables included age entered as a linear variable, cohabitation status as: single or living with partner, children living at home as: none, 1, 2–5 and degree of urbanization as capital area, capital suburban area, provincial cities and rural areas.

In Denmark, organized screening programs were initiated in Copenhagen municipality, Funen County, and Frederiksberg municipality in 1991, 1993 and 1994, respectively. Altogether, 100 000 women aged 50–69 years are covered in the three programs, equivalent to some 20% of the total Danish female population in that age group (Jensen *et al*, 2005). We did not have information on whether individual women had been screened; however, we created a variable ‘mammography screening available’ denoting whether the woman had lived in an area where screening had been available. The screening variable was set as 1, if the woman underwent breast surgery in an area offering mammography screening in the relevant period and age group, and set to 0 otherwise.

### Comorbid disorders

All diagnoses of somatic diseases other than breast cancer were obtained by linking the personal identification number to the files of the Danish National Patient Registry (NPR). Since 1977, the NPR has retained key information on all hospitalizations in Denmark, including the personal identification number of the patient, the date of discharge and up to 20 diagnoses and surgical procedures performed during the hospital stay (Andersen *et al*, 1999). Diagnoses were coded according to a modified five-digit Danish version of the International Classification of Diseases (ICD-8) during 1977–1993; hereafter the ICD-10 was used. By linkage to the NPR, we obtained a full history of diseases leading to hospitalization or outpatient visits for each cohort member from 1978 through 2000.

We used the Charlson Index to classify comorbid disorders, as measured by hospitalisations with the diseases in question from 1979 through to 6 months prior to the breast cancer diagnosis. This scale provides an overall score of comorbidity based on a composite of values weighted by level of severity assigned for a total of 19 selected conditions. Scores for most conditions range from 1 to 3 (Charlson *et al*, 1987). Using these severity weights, the overall comorbidity score is based on the sum of the scores for the individual conditions and the scores were grouped 0, 1 and 2 or more.

### Statistical methods

Logistic regression models were developed to examine the simultaneous influence of all socioeconomic and demographic factors of interest on the likelihood of being diagnosed with a high-risk breast cancer using the procedure PROC LOGISTIC in SAS 9.1. on a UNIX platform. Age was entered linearly in the logistic regression model. This is biologically more reasonable than the step functions corresponding to categorization and furthermore, increases the power of the analysis (Greenland, 1995). Our expectation of a higher proportion of high-risk breast cancer among the youngest women was confirmed by a plot of percent high risk *vs* age at diagnosis. We entered age as a linear spline with a knot at 44 years based on the graphical evaluation as well as a Wald test (*P*<0.0001). Tests for interaction (effect modification) between covariates were performed for one pair of covariates at a time using the Wald test statistic. No significant interactions between any pair of variables were observed. In order to explore the influence of menopausal status on the association between socioeconomic position and high-risk breast cancer, we stratified the cohort according to menopausal status and ran the logistic regression analyses separately in premenopausal and postmenopausal women.

## RESULTS

By use of the risk group definition, 6007 women were diagnosed with low-risk breast cancers and 23 808 women with high-risk breast cancers. Some 1955 women could not be classified due to unknown tumour size (971 women or 3%) or number of positive lymph nodes (529 women; 2%) and were thus excluded from the analyses. Some 360 women (1%) diagnosed with any tumour prior to the breast cancer according to the files of the NPR were excluded in order to be consistent with the criteria of no previous malignant disease in DBCG. A further 702 women (2%) were excluded due to missing information in one or more of the socioeconomic variables leaving a total of 28 765 women for analyses. All women were categorized as either premenopausal (*N*=11 685) or postmenopausal (*N*=17 080) with six women of unknown menopausal status below age 55 classified as premenopausal and one over age 55 as postmenopausal.

Characteristics of the 28 765 women included in the study population with 5809 (20%) women classified as having low-risk breast cancers and 22 956 (80%) women classified as having high-risk breast cancers are shown in Table 1. Although differences were small, slightly more women aged less than 44 years (19 *vs* 16%), who had basic school or high school education (48 *vs* 44%) or low disposable income (27 *vs* 24%) were diagnosed with high-risk breast cancer. Further, more women with 2 or more children living at home (10 *vs* 7%), lived in rural areas (35 *vs* 30%) and with access to screening (93 *vs* 88%) were diagnosed with high-risk breast cancer.

The multivariate analysis showed that the odds ratio (OR) for being diagnosed with a high-risk breast cancer was reduced by 5% per year up to age 44 years (95% CI: 0.94, 0.97) after which age the risk was constant (Table 2). Further, the risk for being diagnosed with a high-risk breast cancer was reduced with increasing length of education and with increasing disposable income with adjusted OR for women with higher education being 12% reduced (95% confidence interval, 0.80, 0.96) compared to women with basic/high school education only whereas the OR was 22% increased among women in the lowest income group (95% CI, (1.10,1.34) compared to women in the highest income group. Occupation, marital status, number of children living at home, housing status, size of dwelling and comorbidity did not influence the risk for being diagnosed with a high-risk breast cancer. There was an urban–rural gradient, with women living in the rural areas of Denmark having a 10% higher OR of high-risk breast cancer (95% CI: 1.02, 1.18) whereas women living in the capital suburban areas had a 15% lower OR (95% CI: 078, 0.93) than those living in provincial cities. The risk among women living in the capital areas was nonsignificantly reduced (OR, 0.93; 95% CI: 0.84, 1.02). Potential exposure to systematic mammography screening almost halved the risk for being diagnosed with high-risk breast cancer (OR, 0.57; 95% CI: 0.51, 0.63).

In premenopausal women the effect of age on risk of being diagnosed with a high-risk breast cancer was similar to that found in the full analysis, with a 5% reduction per year up until age 44 years after which age the risk was close to constant in both premenopausal and postmenopausal women (Table 3). As there were only 40 postmenopausal women below age 44 years, no risk estimate for that group is provided. Further, there was an effect of education, disposable income and urbanicity on the risk for high-risk breast cancer in postmenopausal women. The risk estimates among premenopausal women also tended to be reduced by increasing education, income and urbanicity, although to a lesser degree and failing to reach statistical significance. Access to screening was protective, regardless of menopausal status.

## DISCUSSION

This population-based Danish study shows an increased risk for being diagnosed with a high-risk breast cancer with shorter education, with lower disposable income, with a residence in rural areas, and with having no access to organized mammography screening. Apart from the access to screening, these effects of social inequality were significant only for postmenopausal breast cancers.

The strengths of this study include the unselected cohort of breast cancer patients covering the entire Danish nation and the possibility to define disease progression at the time of diagnosis by four clinical criteria based on information from a nationwide clinical database on breast cancer in Denmark. Further, all information on the socioeconomic and other variables has been collected prospectively and uniformly for administrative purposes independently of our study hypotheses, thus eliminating recall bias and information bias. The tradition for administrative registration in Denmark further enabled us to identify partners and children living at home and thereby adjust disposable income by number of persons in the household.

As expected, we find that access to mammography screening is an important factor for breast cancer stage at diagnosis. Mammography screening is not available throughout the country and furthermore, attendance rates have been reported to average 66% in Copenhagen and 84% in Funen through screening rounds 1–3 (1991–1993, 1993–1995, 1995–1997 in Copenhagen and 1993–1995, 1996–1997 and 1998–1999 in Funen, respectively) (Lynge, 1998; Njor *et al*, 2003), and thus, there might be a social inequality in those who attend these programs. Recent data from the UK indicated a stronger effect of deprivation on disease progression, both measured by stage and by grade in women with access to screening than those not exposed (Adams *et al*, 2004) but, in this study, we did not have screening details for individual women.

We measured socioeconomic position by education, occupation, income and housing acknowledging that each of these indicators measured different although often related aspects. Although the education level might have undergone considerable changes in the women included in our study with more older women in the early study period attaining only basic school education, the similar trends in both pre- and postmenopausal women by education indicate that the categorisation captures an effect probably related to knowledge and skills that in turn affects cognitive functioning (Galobardes *et al*, 2006) and perhaps results in a higher degree of health awareness, better perception of breast related symptoms and less delay in seeking medical care. The possibility of using disposable income based on the household income and adjusted for number of dependent persons must be considered as a good indicator of material living standards, reflecting what the women could actually spend (Galobardes *et al*, 2006). Although most breast cancers are probably diagnosed before generalised symptoms occur, reverse causation cannot be ruled out. However, for a factor such as education, which in most cases would have been attained years prior to breast cancer diagnosis, reverse causation probably plays little role. In the case of income, a factor presumably more sensitive to health changes, we used disposable household income, which probably is a more robust indicator, thus rendering reverse causation less likely.

The social inequality at diagnosis observed in our study contrasts with the increasing risk for breast cancer with increasing education (Danø *et al*, 2003) and occupational group (Dano *et al*, 2004) in Denmark. It remains unclear whether the reason for the disparity by risk-group is delay in diagnosis or differing biology of cancers in the groups with less education and income compared with more advantaged groups. A recent study of UK cancer patients has shown that delay of diagnosis and treatment was longer for lower social class groups than higher social class groups and this was also apparent for breast cancer (Neal and Allgar, 2005). This delay might be a result of lower levels of knowledge regarding significant symptoms and as a result of poorer access to services. The Danish National Health Service provides tax-supported health care for all inhabitants of the country including free access to hospitals, public clinics and general practitioners in addition to reduced fees for most prescription medications. Thus, in theory, there should be no financial barriers to obtaining health care that could explain the social inequality in the likelihood of being diagnosed as high-risk patients. The urban-rural gradient observed in disease progression at diagnosis is also opposite that observed in incidence in Denmark (Ewertz, 1993) and might represent a different degree of delay by rural patients, providers or both, although we know of no relevant Danish study.

We included marital status (Osborne *et al*, 2005) and comorbidity (Satariano and Ragland, 1994; Schrijvers *et al*, 1997) in the analyses because of previous observations that these factors influenced stage, but did not find any such effect on disease progression among Danish breast cancer patients.

The social inequality in risk group of breast cancer by education and disposable income were mainly in postmenopausal women, although similar patterns of increased progression of tumour at diagnosis with shorter education or lower income were observed among the premenopausal women as well. One interpretation of this might be that the more aggressive breast cancers which are characterized by young age at manifestation and genetic predisposition which are predominantly found among premenopausal women are less influenced by the effect of social deprivation on tumour progression.

This population based study shows that less education, disposable income, and degree of urbanization increase the risk of being diagnosed with a high-risk breast cancer in Denmark and that access to mammography screening reduces this risk. Apart from access to mammography screening, these associations seemed stronger in postmenopausal women, suggesting that a subgroup of aggressive premenopausal breast cancers are less influenced by the effect of socioeconomic position on tumour progression at time of diagnosis.

## Figures and Tables

**Table 1:** Descriptive characteristics of 28 765 women diagnosed with primary invasive breast cancer below the age of 70 years in Denmark, 1983–1999

	**Low-risk breast cancer (*N*=5809)**		**High-risk breast cancer (*N*=22 956)**	
**Characteristic**	***N***	**%**	***N***	**%**
*Age at diagnosis*				
<44 years	903	16	4350	19
45–49 years	996	17	3620	16
50–54 years	970	17	3884	17
55–59 years	967	17	3717	16
60–64 years	1010	17	3751	16
65–69 years	963	17	3634	16
				
*Year of diagnosis*				
1983–1987	1314	19	5569	81
1988–1992	1662	20	6561	80
1993–1997	1936	20	7513	80
1998–1999	897	21	3312	79
				
*Menopausal status*				
Premenopausal	2274	39	9411	41
Postmenopausal	3535	61	13 545	59
				
*Highest attained education*				
Basic school/high school	2565	44	10 905	48
Vocational training	1742	30	6621	29
Higher education	1123	19	3991	17
Unknown	379	7	1439	6
				
*Occupation*				
Higher functionary	1072	18	4071	18
Lower functionary	1014	17	4047	18
Skilled worker	378	7	1575	7
Unskilled worker	677	12	2907	13
Unemployed or housewife	869	15	3459	15
Pensioner	1799	31	6897	30
				
*Disposable household income* a				
–99 999 DKK	1369	24	6099	27
100 000–129 999 DKK	1482	26	6022	26
130 000–164 999 DKK	1309	23	5203	23
165 000–DKK	1649	28	5632	25
				
*Cohabiting*				
Living with partner	4128	71	16 571	72
Single	1681	29	6385	28
				
*Children living at home* b				
0	4735	82	18126	79
1	659	11	2641	11
2–5	415	7	2189	10
				
*Housing status*				
Owner-occupied	3719	64	15 250	66
Rental	2090	36	7706	34
				
*Size of dwelling*				
0–99 m^2^	2193	38	8474	37
100–124 m^2^	1168	20	4814	21
125–149 m^2^	1003	17	4083	18
⩾150 m^2^	1445	25	5585	24
				
*Degree of urbanicity*				
Capital areas	944	16	2901	13
Suburbs	916	16	3175	14
Provincial cities	2197	38	8930	39
Rural areas	1752	30	7950	35
				
*Mammography screening*				
Available^c^	5087	88	21 322	93
Not available	722	12	1634	7
				
*Comorbidity* d				
0	5324	92	21 132	92
1	384	6	1384	6
2+	101	2	440	2

Low-risk breast cancer: tumour size <2.0 cm, negative axillary lymph nodes, low histologic grade and oestrogen receptor positive; High-risk breast cancer: tumour size >2.0 cm, positive axillary lymph nodes, high histologic grade and oestrogen receptor negative.

a Household disposable income in Danish crowns (DKK) after taxation and interest adjusted for number of persons in household.

b Children aged 0–17 years.

c Mammography screening was offered to all women aged 50–69 years living in Copenhagen municipality from 1991, Frederiksberg municipality from 1992 and Funen County from 1994.

d Presence of these disorders as defined in Charlson index was defined as an in- or outpatient contact with one of the below diagnosis from 1978 to half a year prior to the diagnosis of breast cancer; Score 1: myocardial infarction, congestive heart failure, peripheral vascular disease, cerebrovascular disease, dementia, chronic pulmonary disease, connective tissue disease, ulcer disease, mild liver disease, diabetes type1, diabetes type2; Score 2: hemiplegia, moderate to severe renal disease, diabetes with end organ damage type1, diabetes with end organ damage type2; Score 3: moderate to severe liver disease; Score 6: AIDS; All cancer related diagnoses were excluded since first primary cancers only are in DBCG.

**Table 2:** Adjusted odds ratios (including 95% CI) of high-risk breast cancer in a cohort of 28 765 women diagnosed with breast cancer below the age of 70 years in Denmark, 1983–1999

	**Odds ratio**	**95% confidence limits**	***P*-value**
*Age*			<0.0001
–44 years (per year)	0.95	0.94, 0.97	
45–69 years (per year)	1.003	0.997, 1.009	
			
*Highest attained education*			0.0060
Basic school/high school	1		
Vocational training	0.92	0.85, 0.98	
Higher education	0.88	0.80, 0.96	
Unknown	0.86	0.76, 0.98	
			
*Occupation*			0.5249
Higher functionary	1.04	0.93, 1.16	
Lower functionary	1.07	0.96, 1.20	
Skilled worker	1.14	0.99, 1.32	
Unskilled worker	1.06	0.94, 1.19	
Unemployed	1.01	0.91, 1.12	
Pensioner	1		
			
*Disposable income* a			0.0020
–99 999 DKK	1.22	1.10, 1.34	
100 000–129 999 DKK	1.12	1.02, 1.23	
130 000–164 999 DKK	1.11	1.02, 1.21	
165 000–DKK	1		
			
*Cohabiting status*			0.54
Living with partner	1		
Single	0.98	0.91, 1.05	
			
*Children living at home* b			0.18
0	1		
1	0.93	0.84, 1.04	
2–5	1.05	0.92, 1.21	
			
*Housing status*			0.22
Owner-occupied	1		
Rental	0.95	0.88, 1.03	
			
*Size of dwelling*			0.35
0–99 m^2^	1		
100–124 m^2^	0.99	0.91, 1.08	
125–149 m^2^	0.97	0.88, 1.07	
150–m^2^	0.92	0.84, 1.02	
			
*Degree of urbanicity*			<0.001
Capital area	0.93	0.84, 1.02	
Suburban area	0.85	0.78, 0.93	
Provincial cities	1		
Rural areas	1.10	1.02, 1.18	
			
*Mammography screening*			<0.001
Not available	1		
Available^c^	0.57	0.51, 0.63	
			
*Comorbidity* d			
0	1		0.27
1	0.94	0.83, 1.06	
2+	1.14	0.92, 1.43	

High-risk breast cancer: tumor size >2.0 cm, positive axillary lymph nodes, high histologic grade and estrogen receptor negative; *P*-values from Wald's test.

a Household disposable income in Danish crowns (DKK) after taxation and interest adjusted for number of persons in household.

b Children aged 0–17 years.

c Mammography screening was offered to all women aged 50–69 years living in Copenhagen municipality from 1991, Frederiksberg municipality from 1992 and Funen County from 1994.

d Presence of these disorders as defined in Charlson index was defined as an in- or outpatient contact with one of the below diagnosis from 1978 to half a year prior to the diagnosis of breast cancer; Score 1: myocardial infarction, congestive heart failure, peripheral vascular disease, cerebrovascular disease, dementia, chronic pulmonary disease, connective tissue disease, ulcer disease, mild liver disease, diabetes type1, diabetes type2; Score 2: hemiplegia, moderate to severe renal disease, diabetes with end organ damage type1, diabetes with end organ damage type2; Score 3: moderate to severe liver disease; Score 6: AIDS; All cancer related diagnoses were excluded since first primary cancers only are in DBCG.

**Table 3:** Adjusted odds ratio (with corresponding 95% confidence limits) of high-risk breast cancer among 11 685 premenopausal and 17 080 postmenopausal women diagnosed with breast cancer in Denmark, 1983–1999

	**Premenopausal women (*N*=11 685)**		**Postmenopausal women (*N*=17 080)**	
	**Odds ratio**	**95% confidence limits**	**Odds ratio**	**95% confidence limits**
*Age*				
–44 years (per year)	0.95	0.93,0.96		
45–69 years (per year)	1.01	1.00,1.03	0.99	0.98,1.00
	*P-value <0.001*		*P-value=0.04*	
				
*Highest attained education*				
Basic school/high school	1		1	
Vocational training	0.92	0.82,1.04	0.92	0.84,1.01
Higher education	0.95	0.82,1.09	0.83	0.73,0.94
Unknown	0.80	0.58,1.11	0.90	0.78,1.04
	*P-value=0.37*		*P-value=0.019*	
				
*Occupation*				
Higher functionary	1.00	0.79,1.26	1.03	0.90,1.19
Lower functionary	1.12	0.89,1.40	0.98	0.85,1.14
Skilled worker	1.14	0.88,1.48	1.10	0.91,1.34
Unskilled worker	1.03	0.82,1.29	1.10	0.94,1.28
Unemployed	1.04	0.82,1.31	0.97	0.87,1.10
Pensioner	1		1	
	*P-value=0.58*		*P-value=0.64*	
				
*Disposable income* a				
–99 999	1.16	0.98,1.37	1.27	1.12,1.44
100 000–129 999	1.05	0.91,1.21	1.18	1.04,1.33
130 000–164 999	1.09	0.96,1.25	1.12	1.00,1.26
165 000–	1		1	
	*P-value=0.30*		*P-value=0.003*	
				
*Cohabiting status*				
Living with partner	1		1	
Single	0.94	0.82,1.07	1.01	0.92,1.10
	*P-value=0.35*		*P-value=0.88*	
				
*Children living at home* b				
0	1		1	
1	0.94	0.83,1.07	1.29	0.96,1.72
2–5	1.13	0.96,1.33	0.70	0.39,1.25
	*P-value=0.06*		*P-value=0.11*	
				
*Housing status*				
Owner-occupied	1		1	
Rental	0.93	0.81,1.06	0.97	0.88,1.07
	*P-value=0.27*		*P-value=0.55*	
				
*Size of dwelling*				
0–99 m^2^	1		1	
100–124 m^2^	0.98	0.84,1.13	1.00	0.90,1.12
125–149 m^2^	0.92	0.79,1.08	1.01	0.88,1.14
150–m^2^	0.92	0.79,1.07	0.93	0.82,1.05
	*P-value=0.62*		*P-value=0.52*	
				
*Degree of urbanicity*				
Capital area	0.96	0.82,1.13	0.92	0.81,1.05
Suburban area	0.93	0.81,1.08	0.80	0.72,0.90
Provincial cities	1		1	
Rural areas	1.06	0.95,1.18	1.13	1.02,1.24
	*P-value=0.38*		*P-value <0.001*	
				
*Mammography screening available*				
Not available	1		1	
Available^c^	0.65	0.47,0.90	0.54	0.49–0.61
	*P-value=0.009*		*P-value <0.001*	
				
*Comorbidity* d				
0	1		1	
1	0.86	0.67,1.10	0.96	0.83,1.10
2–	0.94	0.55,1.59	1.19	0.93,1.52
	*P-value=0.48*		*P-value=0.30*	
				

High-risk breast cancer: tumor size >2.0 cm, positive axillary lymph nodes, high histologic grade and estrogen receptor negative; *P*-values from Wald's test.

a Household disposable income in Danish crowns (DKK) after taxation and interest adjusted for number of persons in household.

b Children aged 0–17 years.

c Mammography screening was offered to all women aged 50–69 years living in Copenhagen municipality from 1991, Frederiksberg municipality from 1992 and Funen County from 1994.

d Presence of these disorders as defined in Charlson index was defined as an in- or outpatient contact with one of the below diagnosis from 1978 to half a year prior to the diagnosis of breast cancer; Score 1: myocardial infarction, congestive heart failure, peripheral vascular disease, cerebrovascular disease, dementia, chronic pulmonary disease, connective tissue disease, ulcer disease, mild liver disease, diabetes type1, diabetes type2; Score 2: hemiplegia, moderate to severe renal disease, diabetes with end organ damage type1, diabetes with end organ damage type2; Score 3: moderate to severe liver disease; Score 6: AIDS; All cancer related diagnoses were excluded since first primary cancers only are in DBCG.
